# Cardiac MRI detection of infarct size reduction with hypothermia in porcine ischemia reperfusion injury model

**DOI:** 10.1186/1532-429X-17-S1-P115

**Published:** 2015-02-03

**Authors:** Rajesh Dash, Atsushi Tachibana, Yoshiaki Mitsutake, Fady Dawoud, Fumiaki Ikeno, Jennifer K Lyons, Michael V McConnell, Alan Yeung, Uday Illindala, Phillip Yang

**Affiliations:** Stanford University, Stanford, CA USA; Zoll, Inc, San Jose, CA USA

## Background

Hypothermia has been shown to provide cardiac protection after a STEMI insult in prior animal and post-hoc human studies. However, the influence of pre-reperfusion temperature on myocardial infarct size is not fully characterized. We employed cardiac MRI to determine the impact of moderate hypothermia (32C) upon infarct size in a porcine ischemia-reperfusion injury (IR) model using an endovascular cooling catheter.

## Methods

Nineteen (19) adult Yorkshire swine underwent 60-minute ischemia reperfusion (IR) injury (left anterior descending coronary artery) with intravascular cooling administered *before reperfusion*. The swine were split into 3 groups defined by core temperature before reperfusion: a) 32C (n=6) b) 35C (n=7) and c) 38C (control, n=6). Cardiac MRI (CMR, 3T GE Inc.) was performed at day 0 and 6 days post-IR for delayed gadolinium enhancement scar volumes (DEMRI, d6 only), which were analyzed using CMR42 software (Circle CVI, Inc). Hearts were stained for scar and area at risk (AAR) by TTC. Semi-automatic infarct quantification was achieved using two methods: full width half maxium (FWHM), 5 and 6 standard deviations above mean signal (5SD and 6SD).

## Results

**Figure 1:**
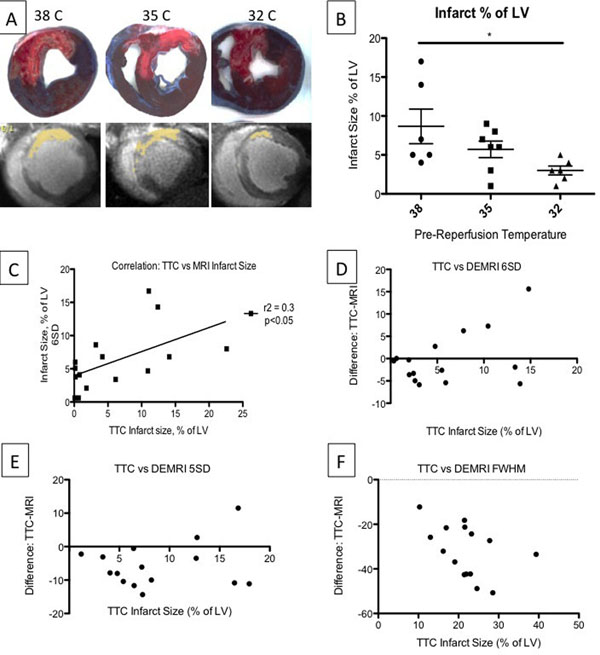
**Moderate Hypothermia Reduces Infarct size.** (A) TTC (top: scar tissue is bright white), DEMRI (bottom: yellow areas are 6SD above mean signal intensity = scar tissue). Note visibly reduced injury/infarct in 32C group; (B) Only 32C hearts (n=6) exhibited significant reduction in infarct size (as % of LV) vs. 38C (n=6). 35C (n=7); (C) Infarct size by CMR best correlated with TTC histopathological scar using 6SD threshold method. (D-F) Bland-Altman plots show good agreement between TTC Infarct size and 6SD, but poor agreements with 5SD and FWHM Infarct size.

On day 6, hypothermia significantly reduced infarct size when compared to 38C (Figure 1A,B). This reduction was most accurately quantified using the highest threshold DEMRI analysis, 6SD (TTC & MRI-derived infarct size at 6SD, correlation r=0.55, p<0.05, Figure 1C). 5SD and FWHM significantly overestimated infarct size when compared to TTC histopathologic infarct sizing, as shown in Bland Altman analyses (Figure 1D-F).

## Conclusions

Pre-reperfusion hypothermia reduces infarct size compared to 38C. Notably, the small infarct sizes observed in 32C were best assessed using a high threshold method of 6SD, and were largely overestimated by 5SD and FWHM methods. Longer-term studies are required to fully evaluate the impact specific pre-reperfusion temperature reduction upon infarct size and long-term cardiac recovery after STEMI.

## Funding

NIH/NHLBI K08 (RD) Zoll, Inc.

